# Tropical forest recovery from logging: a 24 year silvicultural experiment from Central Africa

**DOI:** 10.1098/rstb.2012.0302

**Published:** 2013-09-05

**Authors:** Sylvie Gourlet-Fleury, Frédéric Mortier, Adeline Fayolle, Fidèle Baya, Dakis Ouédraogo, Fabrice Bénédet, Nicolas Picard

**Affiliations:** 1 BSEF, CIRAD, 34398 Montpellier, France; 2 Agro Bio-Tech, Université de Liège, 5030 Gembloux, Belgium; 3 Ministère des Eaux, Forêts, Chasse et Pêche, BP 3314 Bangui, Central African Republic; 4 BSEF, CIRAD, BP 4035 Libreville, Gabon

**Keywords:** moist semi-deciduous forests, silviculture, permanent sample plots, above-ground biomass, timber species

## Abstract

Large areas of African moist forests are being logged in the context of supposedly sustainable management plans. It remains however controversial whether harvesting a few trees per hectare can be maintained in the long term while preserving other forest services as well. We used a unique 24 year silvicultural experiment, encompassing 10 4 ha plots established in the Central African Republic, to assess the effect of disturbance linked to logging (two to nine trees ha^−1^ greater than or equal to 80 cm DBH) and thinning (11–41 trees ha^−1^ greater than or equal to 50 cm DBH) on the structure and dynamics of the forest. Before silvicultural treatments, above-ground biomass (AGB) and timber stock (i.e. the volume of commercial trees greater than or equal to 80 cm DBH) in the plots amounted 374.5 ± 58.2 Mg ha^−1^ and 79.7 ± 45.9 m^3^ ha^−1^, respectively. We found that (i) natural control forest was increasing in AGB (2.58 ± 1.73 Mg dry mass ha^−1^ yr^−1^) and decreasing in timber stock (−0.33 ± 1.57 m^3^ ha^−1^ yr^−1^); (ii) the AGB recovered very quickly after logging and thinning, at a rate proportional to the disturbance intensity (mean recovery after 24 years: 144%). Compared with controls, the gain almost doubled in the logged plots (4.82 ± 1.22 Mg ha^−1^ yr^−1^) and tripled in the logged + thinned plots (8.03 ± 1.41 Mg ha^−1^ yr^−1^); (iii) the timber stock recovered slowly (mean recovery after 24 years: 41%), at a rate of 0.75 ± 0.51 m^3^ ha^−1^ yr^−1^ in the logged plots, and 0.81 ± 0.74 m^3^ ha^−1^ yr^−1^ in the logged + thinned plots. Although thinning significantly increased the gain in biomass, it had no effect on the gain in timber stock. However, thinning did foster the growth and survival of small- and medium-sized timber trees and should have a positive effect over the next felling cycle.

## 1. Introduction

The forests of Central Africa (186.9 million ha, comprising 90% of lowland forests [1]) are facing increasing anthropogenic pressure. Logging in particular, has been accused of promoting human settlement and the bushmeat trade and of degrading forests ([2–4], but see [5]). Indeed, for a long time, logging has been a poorly regulated mining-type activity, entirely dependent on economic constraints, with little regard for sustainability. Typical harvesting is highly selective (one to two trees ha^−1^ and sometimes less at each passage [6,7]; however, in many places, market opportunities have prompted loggers to make repeated entries, thus depleting the stock of adult commercial trees, as well as causing considerable damage to the stands [8].

With the increasing worldwide concern for the fate of tropical forests [9], the need for sustainable management and conservation has been highlighted. From 1990 to 2002, most countries in Central Africa redefined their forestry laws, making management plans for production forests mandatory [10]. As a result, over 14 million ha of forests now have a management plan, out of 44 million ha allocated to logging in long-term concessions [11].

Management plans are supposed to be consistent with sustainable forest management as defined by the International Tropical Timber Organization. They are supposed to ensure ‘the production of a continuous flow of desired forest products and services without undue reduction of (the forest's) inherent values and future productivity and without undue undesirable effects on the physical and social environment’ [12, p. 34]. Whether timber can be sustainably produced has been a highly controversial topic ([3,13,14] versus [8,15,16]). A recent review [17] concluded that, in spite of recognized adverse effects [3], once-logged forests retained substantial timber stocks, carbon stocks and biodiversity and that conservation efforts should be focused on improving logging, rather than prohibiting it.

Central to the controversy are the impacts of selective logging—and more generally the potential effects of silviculture—on the structure, composition and dynamics of tropical forests. There is extensive literature on these topics elsewhere in the tropics, but very few studies have been published on Central Africa [7,16,18–22], and even fewer rely on longitudinal experiments. In this paper, we present data and results from a unique long-term silvicultural experiment in the Central African Republic (CAR). Permanent plots were established in a semi-deciduous moist forest in 1982, and two silvicultural treatments of increasing intensity (logging, logging + thinning) were applied to a subset of the plots. In a previous study, we showed that the treatments had little impact on the floristic composition of the forest and that thinning promoted growth and survival of non-pioneer species to the detriment of pioneer species [22]. Here, we quantified the response of above-ground biomass (AGB) and timber stock to the treatments over a period of 24 years, i.e. close to the duration of one complete felling cycle (25–30 years in the region). We examined the speed of the forest recovery in relation to the logging intensity, and the effect of thinning on recovery. We then discussed the implications of our results for forest management.

## 2. Material and methods

### (a) Study site

M'Baïki experimental site (3°90′ N, 17°93′ E) was established in 1982 in protected forests with no recent logging history. Average annual rainfall is 1739 mm (1981–2008) with a three- to four-month dry season (November/December–February). Annual average monthly temperature is 24.9°C (19.6–30.2°C, 1981–1989). The site is located on a large plateau (500–600 m a.s.l.), and soils are deep ferralitic soils, classified as acrisols [23]. The vegetation is semi-deciduous moist forest of the Guineo-Congolian domain [24], and the canopy is dominated by species from the Cannabaceae, Myristicaceae and Meliaceae families [22].

### (b) Experimental design

Ten 4 ha (200 × 200 m) permanent plots were established within a 10 km radius circle. Each 4 ha plot (core zone) was composed of four 1 ha subplots and surrounded by a 50 m wide buffer zone. All trees with a diameter greater than or equal to 10 cm at breast height (DBH) or 50 cm above buttresses (4m50 since 2007; see electronic supplementary material, method section S1) were individually marked, geo-referenced and identified inside the subplots. The plots have been yearly monitored since 1982 (except in 1997, 1999 and 2001).

The 10 plots were assigned to three different silvicultural treatments according to a random block design: control (three plots), logging (three plots), logging + thinning (four plots). The treatments were applied both to the core and the buffer zones to avoid edge effects. Between 1984 and 1985, seven 4 ha plots were thus selectively logged, i.e. the harvested trees belonged to 16 timber species and were greater than or equal to 80 cm DBH (list in electronic supplementary material, table S1). The skid trails were planned after felling to minimize the distance between logs and landings, and skidding involved a Caterpillar D8 tractor and two Caterpillar 528 rubber-tyred skidders [25]. Between 1986 and 1987, four of the logged plots were additionally thinned to increase light penetration in the understory: all trees greater than or equal to 50 cm DBH from non-timber species were poison-girdled (arboricide Triclopyr—GARLON 4E 480 g l^−1^ ester butylglycol, injected inside circular girdling cuts), and all lianas were removed.

At a 4 ha scale, timber trees are not evenly distributed; as a result, the logging intensity and induced disturbance were highly heterogeneous inside the plots (details in [22]). In the analyses, we thus used the 40 subplots (100 × 100 m) as statistical units in order to decrease intra-unit variability—and to better capture the disturbance effect—while limiting inter-unit spatial autocorrelation.

### (c) Above-ground biomass and timber stock

The AGB (kg; [26]) and over-bark volume (*v*_s_ in m^3^; [27]) of a tree belonging to a species s were calculated as follows:2.1
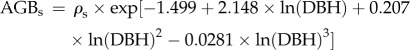


and2.2
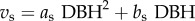
with DBH in centimetres (2.1) or in metres (2.2), **ρ**_s_ denotes specific wood density, extracted from CIRAD's database on wood properties [28], *a*_s_ and *b*_s_ denote specific regression coefficients that follow from the form and taper of the stem (see the electronic supplementary material, table S1). All trees with raised points of measurement were corrected to get DBH values (see the electronic supplementary material, section Method S1).

We summed the AGB_s_ values of trees greater than or equal to 10 cm DBH to get the AGB at subplot level. To get the timber stock (V_80_), we summed the *v*_s_ values of trees greater than or equal to 80 cm DBH belonging to 39 timber species, 16 of which had harvestable trees in 1984 (list in electronic supplementary material, table S1). Most of the trees felled belonged to the following three light-demanding species: *Triplochiton scleroxylon* K. Schum., Malvaceae (‘ayous’, 35%), *Entandrophragma cylindricum* (Sprague) Sprague, Meliaceae (‘sapelli’, 33%) and *Terminalia superba* Engl. and Diels, Combretaceae (‘limba’, 7%). *Entandrophragma cylindricum* and *T. scleroxylon* are the second and third most logged species in Central Africa [11].

We also calculated, for each subplot: (i) the changes over time since 1982 in AGB and V_80_; (ii) the annualized net change in AGB and V_80_ after treatments, i.e. between 1987 (right after treatment) and 2011; (iii) the recovery rates of AGB and V_80_ as the ratios between the amounts lost during treatments (1984–1987) and the amounts gained between 1987 and 2011, multiplied by 100 (table 1 and electronic supplementary material, table S2).

**Table 1.** Description of the silvicultural treatments, disturbance intensity and evolution of the forest structure and changes in biomass and timber stock in each subplot. G_L: basal area of the trees logged in 1984 (number of trees in parentheses). G_P: basal area of the trees poison-girdled in 1986 (number of trees in parentheses). Total G lost: sum of G_L, G_P and the basal area broken during logging operations. G: basal area of living trees greater than or equal to 10 cm DBH, AGB: above-ground biomass, V_80_: timber stock, i.e. standing volume of the commercial trees (DBH ≥ 80 cm, belonging to 39 timber species). The annual change of AGB (resp. V_80_) was calculated as (AGB^2011^–AGB^1987^)/24. The percentage of AGB (resp. V_80_) recovered was calculated as (AGB^2011^–AGB^1987^)/(AGB^1984^–AGB^1987^) × 100. The same information given for *N* (total number of living trees greater than or equal to 10 cm DBH) and G (total basal area) in electronic supplementary material, table S2.

plot identification		disturbance intensity			AGB (Mg ha^−1^)			V_80_ (m^3^ ha^−1^)		
subplot^a^	treatment^b^	G_L^c^ (m^2^ ha^−1^)	G_P^c^ (m^2^ ha^−1^)	total G lost (m^2^ ha^−1^)	1984 value	annual change (1987–2011)	% recovered in 2011	1984 value	annual change (1987–2011)	% recovered in 2011
111	L	6.46 (5)	0	8.02	349.8	4.39	95.2	88.4	0.908	29.4
112	L	1.62 (2)	0	1.97	349.0	2.88	115.8	43.1	0.050	4.0
113	L	5.07 (4)	0	10.67	399.5	3.27	51.9	82.6	0.289	11.7
114	L	0	0	1.57	259.0	5.38	—	13.0	1.417	—
121	L+T	3.47 (2)	6.77 (24)	10.50	359.8	9.14	152.8	45.6	0.855	77.8
122	L+T	0	3.94 (17)	4.03	258.9	8.33	510.9	7.6	0.963	—
123	L+T	1.49 (1)	2.65 (11)	5.13	286.0	7.05	193.9	30.6	0.279	29.0
124	L+T	1.03 (1)	8.66 (25)	9.90	315.3	6.50	118.7	27.7	0.251	63.0
131	C	0	0	0	313.7	3.46	—	19.5	0.831	—
132	C	0	0	0	400.4	4.44	—	60.9	0.586	—
133	C	0	0	0	374.1	3.28	—	28.9	0.311	—
134	C	0	0	0	433.4	4.80	—	59.7	1.742	—
141	L	7.95 (7)	0	12.19	410.9	4.94	88.8	140.4	1.186	28.2
142	L	2.35 (3)	0	5.36	322.4	6.38	266.5	36.0	1.550	103.4
143	L	4.21 (4)	0	7.40	338.7	4.95	141.8	69.8	0.607	24.7
144	L	5.30 (4)	0	7.88	451.5	6.88	122.3	69.7	0.668	24.7
151	L+T	2.66 (2)	3.7 (17)	7.53	266.8	9.80	290.2	47.7	0.926	81.4
152	L+T	3.44 (3)	7.27 (27)	11.63	331.8	9.15	156.0	59.7	2.319	120.4
153	L+T	5.20 (4)	8.39 (25)	15.92	397.8	10.22	118.5	74.2	1.203	38.9
154	L+T	3.52 (2)	7.77 (16)	12.84	430.1	10.30	121.2	62.0	1.608	108.3
161	C	0	0	0	413.7	2.31	—	138.6	−0.488	—
162	C	0	0	0	392.1	3.95	—	67.4	−0.135	—
163	C	0	0	0	426.5	−0.69	—	184.4	−4.340	—
164	C	0	0	0	335.6	3.45	—	51.1	0.975	—
211	L	10.64 (9)	0	12.95	475.4	4.71	60.8	140.7	0.392	8.0
212	L	5.52 (5)	0	7.42	365.3	5.58	127.8	106.0	0.093	2.9
213	L	3.40 (2)	0	5.26	396.7	3.29	169.3	99.0	1.228	59.4
214	L	6.95 (7)	0	8.96	380.7	5.17	109.9	89.7	0.661	18.8
221	L+T	5.78 (5)	4.62 (22)	13.45	385.6	7.27	92.7	107.8	−0.406	−13.6
222	L+T	5.64 (4)	5.42 (16)	12.32	386.8	8.37	117.0	90.8	1.927	76.1
223	L+T	8.62 (7)	3.03 (14)	16.48	422.2	6.87	75.6	128.3	0.497	13.2
224	L+T	5.07 (3)	9.00 (26)	14.84	464.8	8.25	90.0	81.5	1.344	65.1
231	L+T	6.36 (5)	1.89 (11)	11.15	405.5	7.44	84.7	185.5	0.671	12.1
232	L+T	10.26 (7)	4.37 (21)	17.54	389.7	6.70	83.1	167.9	0.460	7.6
233	L+T	0	8.19 (41)	8.40	311.2	5.49	92.0	56.9	−0.102	—
234	L+T	0	4.9 (20)	6.02	281.3	7.58	241.0	12.1	0.192	—
241	C	0	0	0	336.4	1.51	—	75.3	−0.304	—
242	C	0	0	0	366.9	1.58	—	110.9	−0.764	—
243	C	0	0	0	374.6	3.07	—	88.5	−0.561	—
244	C	0	0	0	329.0	−0.19	—	84.8	−1.788	—

^a^The first two numbers identify the plot. In each plot, the four subplots are numbered 1 to 4.

^b^C, control; L, logging; L+T, logging+thinning.

^c^Number of trees logged (resp. thinned) in parentheses.

We quantified separately the disturbance intensity caused by logging and thinning, by computing the cumulated basal area of trees logged between 1984 and 1985 (G_L) and trees poison-girdled between 1986 and 1987 (G_P) (table 1 and electronic supplementary material, table S2).

### (d) Statistical analyses

We compared the forest structure in 1982 and 2011 in the control subplots, and we tested the effect of silvicultural treatments on structure, timber stock, recovery rates and annualized net change in biomass and timber stock. Because the subplots shared the same local environment at plot scale, we used linear mixed models to take into account spatial autocorrelation. We also modelled intra-treatment variance when necessary (details in electronic supplementary material, method section S2 and results in table S3).

We tested the additive effects and interactions of several covariates on the annualized net changes after treatments: the remaining living material after treatment (AGB or V_80_ values in 1987), the logging intensity (G_L), the thinning intensity (G_P) and the time elapsed since the end of treatments (*t*). Because the observations had been made on the same subplot over time, and because the subplots shared the same local environment at plot scale, we again used a linear mixed model to take into account both temporal and spatial autocorrelations (details in electronic supplementary material, method section S3).

Statistical analyses were performed with R [29]. Linear mixed models were fitted with lme4 and nlme packages.

## 3. Results

### (a) The initial forest structure and its changes in control plots over time

The initial structure at  M'Baïki was characteristic of old-growth forests, with lots of small trees and few large ones, mainly timber trees, storing a high proportion of biomass.

In 1982, the mean (±s.e.) number of living trees greater than or equal to 10 cm DBH, G, AGB and V_80_ in all subplots (*n* = 40) were N^1982^ = 578.7 ± 42.4 trees ha^−1^, G^1982^ = 32.6 ± 3.9 m^2^ ha^−1^, AGB^1982^ = 374.5 ± 58.2 Mg dry mass ha^−1^, and V_80_^1982^ = 79.7 ± 45.9 m^3^ ha^−1^, respectively. There was no significant difference between the groups of subplots assigned to the three treatments.

In the control subplots, the diameter distribution exhibited a typical reverse J-shaped distribution, with 66.8% of the trees less than 20 cm DBH and only 1.5% of the trees greater than or equal to 80 cm DBH (see the electronic supplementary material, figure S2*a*). Although the number of large trees was low (8.9 ± 2.9 trees ha^−1^ greater than or equal to 80 cm DBH), they stored a large amount (35.9%) of AGB: 137.4 ± 41.6 Mg ha^−1^ (see the electronic supplementary material, figure S2*b*). The 39 timber species represented 21.4% of the number of living trees overall, but 42% of AGB because most of the large trees (71.9% of trees greater than or equal to 80 cm DBH, 68.6% of corresponding AGB) belonged to timber species.

**Figure 1.**
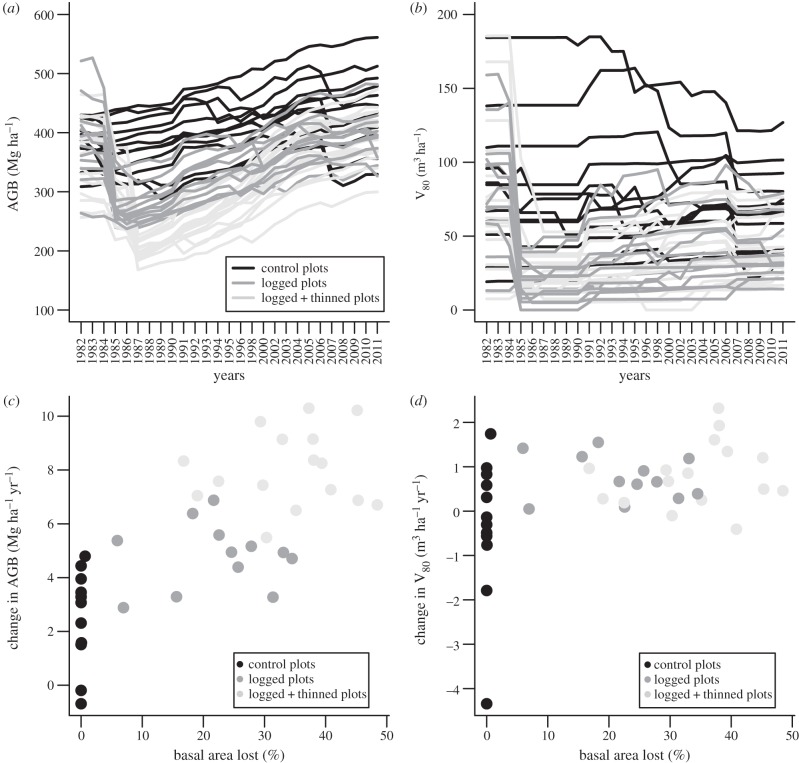
Long-term variations in forest structure and changes in biomass or timber stock (see also electronic supplementary material, figure S3 for N and G). Evolution in (*a*) above-ground biomass and (*b*) timber stock are shown according to the silvicultural treatment. (*c*) Annualized net change in above-ground biomass and (*d*) timber stock are shown according to the disturbance intensity (basal area lost = total G lost × 100/G1984; table 1). The period used to calculate the annualized net changes is 1987–2011.

In 2011, the mean number of living trees, G, AGB and V_80_ in the control subplots (*n* = 12) were N^2011^ = 603.1 ± 66.9 trees ha^−1^, G^2011^ = 36.6 ± 3.6 m^2^ ha^−1^, AGB^2011^ = 441.4 ± 65.7 Mg ha^−1^ and V_80_^1982^ = 71.3 ± 26.8 m^3^ ha^−1^, respectively. N, G and AGB increased between 1982 and 2011 (1.3 ± 2.2 trees ha^−1^ yr^−1^, 0.13 ± 0.13 m^2^ ha^−1^ yr^−1^, 2.04 ± 1.84 Mg ha^−1^ yr^−1^, respectively), whereas V_80_ decreased (−0.37 ± 1.32 m^3^ ha^−1^ yr^−1^). These trends however concealed a high heterogeneity in trajectories among subplots (figure 1*a*). Three of them exhibited a net biomass loss over the whole period (−1.9%, −2.7% and −9%, respectively) because of large trees dying between 2005 and 2008. The most dynamic subplot exhibited a 23.4% increase over the same period. Six subplots, comprising those losing biomass, exhibited a net loss (−8.1 to −56.4%) in timber stock, whereas the most dynamic subplot doubled its stock (figure 1*b*). Diameter distributions changed between 1982 and 2011, with an increase in tree numbers mainly in the intermediate classes (20–30 to 50–60 cm DBH), and an increase in mean AGB in all classes but the smallest one (10–20 cm DBH; electronic supplementary material, figure S2).

### (b) Forest recovery after logging and thinning

Logging removed two to nine trees ha^−1^ (1.5–10.6 m^2^ ha^−1^, 10–134.8 m^3^ ha^−1^ and 15.7–173.1 Mg ha^−1^ in terms of basal area, volume and AGB, respectively), whereas thinning removed 11–41 trees ha^−1^ (0.02–9.0 m^2^ ha^−1^, 0.11–85.7 m^3^ ha^−1^ and 2.68–135.4 Mg ha^−1^). When adding damage to the stands, total loss amounted to 0.02–17.5 m^2^ ha^−1^ (0.11–201.3 m^3^ ha^−1^, 16.2–232 Mg ha^−1^), i.e. 0.05–48.5% of the initial basal area (see the electronic supplementary material, table S2 and figure S4). Four of the treated subplots had no harvestable trees in the core zone but underwent damage from trees harvested in the buffer zone.

Twenty-four years after the end of the silvicultural treatments, mean AGB recovery in the treated subplots had reached 144%, and 63% of the treated subplots had recovered initial AGB (64% and 62% in the logging and logging + thinning treatments respectively). By contrast, mean V_80_ recovery had reached only 41.4% and only 13% of the logged subplots had recovered initial timber stock (9% and 14% in the logging and logging + thinning treatments, respectively; table 1 and figure 1*a,b* and electronic supplementary material, figure S5).

All the subplots that had less than 5 m^2^ ha^−1^ of basal area logged (less than four trees ha^−1^ greater than or equal to 80 cm DBH) recovered 100% of their AGB in 2011, regardless of the additional amount lost by thinning (see the electronic supplementary material, figure S5). The three subplots that had recovered 100% of their V_80_ were a subset of those plots.

We found no significant effect of the treatment type on the level of recovery, be it AGB or V_80_. This suggested a compensating effect between the treatment intensity and the annualized net change after treatment.

### (c) Effect of logging and thinning on changes in biomass and timber stock

The silvicultural treatments significantly increased the gain in AGB, but not in timber stock.

Compared with controls, the gain in AGB almost doubled in logged subplots (from 2.58 ± 1.73 to 4.82 ± 1.22 Mg ha^−1^ yr^−1^) and tripled in logged + thinned subplots (8.03 ± 1.41 Mg ha^−1^ yr^−1^; figure 1*c*). The gain in V_80_ was higher in treated versus control plots (logging: 0.75 ± 0.51 m^3^ ha^−1^ yr^−1^, logging + thinning: 0.81 ± 0.74 m^3^ ha^−1^ yr^−1^, control: −0.33 ± 1.57 m^3^ ha^−1^ yr^−1^; figure 1*d*) because of two control subplots that experienced particularly high mortality losses during the period.

Annual change modelling further evidenced that (table 2): (i) the gain in AGB significantly increased when G_L or G_P increased, and significantly decreased when the time since treatment increased; (ii) the gain in V_80_ significantly decreased when V_80_^1987^ increased, without any effect of the treatment intensity nor of the time since treatment.

**Table 2.** Parameters of the best-selected models among all models including or not random plot and subplot effects (cf. electronic supplementary material, method section S3 and details on full models testing in table S4). AGB, above-ground biomass; V_80_, standing volume of the commercial trees (DBH ≥ 80 cm, belonging to 39 timber species). ∂AGB (resp. ∂V_80_): annual change of AGB (resp. V_80_). G_L and G_P: as in table 1, *t*: time elapsed since the end of treatments (namely 1987, thus varying from 1 to 24). Models including plots and subplots as random effects always exhibited higher BIC values (with very low variance of the random effects) than corresponding models with fixed effects.

fixed terms	estimate	s.e.	*t*-value	Pr(>|*t*|)
variable predicted: ∂AGB (*n* = 838^a^)				
final model (BIC = 5726.73, adjusted *R*^2^ = 0.102)				
(intercept)	7.487	0.735	10.187	<0.001***
G_L	0.234	0.080	2.933	0.003**
G_P	0.551	0.081	6.817	<0.001***
*t*	−0.208	0.034	−6.118	<0.001***
variable predicted: ∂V_80_ (*n* = 838^a^)				
final model (BIC = 4763.32, adjusted-*R*^2^ = 0.017)				
(intercept)	1.178	0.204	5.759	<0.001***
V_80_^1987^	−0.015	0.004	−3.942	<0.001***

^a^Three years were missing: 1997, 1999, 2001 (see §2). ∂AGB and ∂V_80_ were calculated over 2-years periods and divided by two. The theoretical number of observations is thus *n* = 40 (subplots) × (24–3) (number of years considered) = 840. We also deleted two extremely negative values observed for two control subplots in 2007 (subplots 163 and 244).

## 4. Discussion

To the best of our knowledge, there is no comparable study documenting the effects of logging and thinning on Central African moist forests. Almost three decades of observations in 40 ha of initially preserved old-growth forests highlighted that (i) natural forests in the area store high levels of AGB and are still accumulating biomass; (ii) the AGB recovers very fast after logging and thinning, at a rate proportional to the disturbance intensity; and (iii) the timber stock recovers slowly and remains far from initial levels accumulated over long periods. Even if thinning significantly increased the gain in biomass, it had no effect on the gain in timber stock.

### (a) Natural forests in M'Baïki are not at equilibrium

Although initial AGBs in M'Baïki in 1982 (374.5 ± 58.2 Mg ha^−1^) were very close to the values reported across Africa (398.5 ± 111.1 Mg ha^−1^ [30]; 395.7 ± 117.4 Mg ha^−1^ [31]), they tended to be lower than the values obtained in Central African countries (434.4 ± 90.5 Mg ha^−1^, *n* = 36 plots located in Cameroon, the Democratic Republic of Congo and Gabon; electronic supplementary material, figure S6*a,b*). They were however higher than the mean values observed from large-scale inventory plots on acrisols in two concessions of CAR (286.8 ± 104.1 Mg ha^−1^ for DBH ≥ 10 cm, *n* = 329 plots supposedly undisturbed [28]), and from Central African permanent plots on acrisols of the AfriTRON network (338.5 ± 103.9 Mg ha^−1^ for DBH ≥ 10 cm, *n* = 6 plots [31]).

In control plots, AGB grew to 441.4 ± 65.7 Mg ha^−1^ in 2011. This change resulted from a net increase in both tree numbers and basal areas, particularly in medium-sized trees. In contrast to AGB, the gain in AGB tended to be higher in M'Baïki than elsewhere in Central Africa (2.58 ± 1.73 Mg ha^−1^ yr^−1^ in M'Baïki versus 1.86 ± 4.40 Mg ha^−1^ yr^−1^ [30]; electronic supplementary material, figure S6*a,c*). These results suggest that the control plots are not at equilibrium. The observed change might be caused by (i) increasing resource availability, consistent with carbon dioxide fertilization hypotheses and the more widespread increase in forest biomass in Africa and across the tropics [30,32,33]; or (ii) recovery from large-scale ancient disturbances, consistent with the dominance of light-demanding species in the canopy. Similar patterns in African moist forests have been attributed to human occupation and shifting cultivation [34–37], or to severe drought and fire episodes during the past 500 years [38,39]; or (iii) some combination of the two.

### (b) Logged forests quickly recovered their above-ground biomass after disturbance, and thinning fostered this recovery

High levels of living material were removed by the treatments. Logging removed, on average, about fourfold the number of trees and between fivefold and sixfold the volume and AGB currently felled in the region (one to two trees ha^−1^, 10–15 m^3^ ha^−1^, less than 20 Mg ha^−1^ [6,17,20]). Despite high AGB losses from timber extraction, thinning and associated damages, 63% of the treated subplots had recovered initial AGB after 24 years and 85% would recover it within 30 years according to our model (table 2).

The gain in biomass increased with logging and thinning intensity: the higher the amount of basal area lost, the higher the gain, regardless of the level of biomass left after treatment (table 2). As observed elsewhere [40–42], openings increased the growth of remaining trees and the recruitment of new trees, with an increase in the proportion of fast-growing pioneers. In addition, thinning fostered the growth and survival of non-pioneer light-demanding trees including those able to reach the canopy [22]. The increased gain in AGB mainly resulted from the increased growth of these groups of species, combined with reduced mortality losses (see the electronic supplementary material, figure S7). The gain has however been decreasing with time since treatment, which was to be expected, because competition increases as stands accumulate biomass.

Thinning by poison-girdling is a silvicultural practice designed to foster the growth of future crop trees by increasing light availability in forests while limiting damage [43]. In M'Baïki, thinning had an additional positive effect on the gain in AGB regardless of the logging intensity, allowing recovery rates that were identical to those from logging despite higher biomass loss. However, our results showed that, when logging intensity exceeded four trees ha^−1^ (or 5 m^2^ ha^−1^ of basal area), the additional loss owing to thinning delayed full recovery beyond 24 years in most cases (see the electronic supplementary material, figure S5*b*).

Such high recovery rates observed in M'Baïki contrast with observations elsewhere in African forests [16,44–46], where the basal area may require more than 100 years to recover [45]. Differences might be explained by a combination of factors [46,47]: higher soil fertility, presence of a large pool of pioneers—comprising the very fast growing *Musanga cecropioides* R. Br. (Urticaceae), absence of invading climbers, lianas, shrubs or herbs able to arrest succession, absence of elephants and other ground-dwelling animals able to delay succession, as a result of intense hunting by local villagers, and limited soil damage from logging operations. Differences might also result from initial states and the unknown exact intensity of treatments in most cited studies: in some cases, low recovery might merely result from comparisons with non-representative control plots.

### (c) In first-time-logged forests, the timber stock did not recover within one felling cycle and thinning did not foster this recovery

In marked contrast with biomass, the timber stock recovered very slowly after treatments: only 12% of the subplots had recovered their volume in 2011. Neither the logging intensity, nor the thinning intensity stimulated the gain. The V_80_ value in 1987 was the sole variable found to relate with gain (table 2): the higher the timber stock, the lower the gain. This could be explained by some control subplots losing large trees between 1987 and 2011, most of them being light-demanding timber species. No such mortality events were observed in the treated subplots, where most of the large trees had been extracted and the survival of the remaining trees might have thus increased.

In the literature, it has been well established that it is impossible for a first-time-logged tropical forest to recover its timber stock within the usual duration of a felling cycle (synthesis in [17]). Thinning should have improved recovery, but the lack of visible effect on the gain in timber stock appears both disappointing and questioning. Disappointing because the primary rationale of this operation is to promote future crop trees by giving them increasing access to resources [43,48]. Questioning because the positive effects of thinning and/or liberation operations have been evidenced in many places [41,42,49,50].

Thinning did have a high positive effect on timber species populations, but 24 years was not long enough to show this effect on the gain in timber stock: removing non-timber trees mainly benefitted timber trees of intermediate size classes, i.e. smaller than 70 cm DBH, with an increasing effect as diameters decreased (see the electronic supplementary material, figure S8). Given the mean growth rate observed in the 60–70 cm DBH class during the past five years, the positive effect of thinning on timber stock should be detectable within 20–45 years, i.e. in the course of the second felling cycle provided the future crop trees are carefully preserved.

It is worth emphasizing that low levels of logging and overall disturbance cannot guarantee full recovery within a felling cycle, even when completed by thinning (see the electronic supplementary material, figure S5*d*). Full recovery mainly depends on the relative abundance of future crop trees under the diameter cutting limit and thus on the autecology of timber species and the history of their populations on the site [51].

### (d) Consequences for management in the Central African region

M'Baïki long-term experiment highlighted that moist semi-deciduous forests may be resilient to disturbance linked to silvicultural operations, even under high intensity levels. The gain in biomass proved to be particularly high, leading to fast recovery rates. Because the gain in biomass increased with the disturbance intensity, recovery rates were not affected by the quantity of basal area lost. However, logging deeply modified the forest structure by extracting large trees from the canopy, and this group of trees only recovered a small part of its volume in 24 years. Thus, whereas the carbon storage service did not appear at stake, production of a continuous flow of timber, even at low felling intensity, will entail adapting the rules. Indeed, in first-time-logged forests of Central Africa, managers face two major issues: (i) the high level of timber stock in the forest is impossible to recover within a felling cycle even if the length of the cycle is doubled; (ii) the species logged have high light requirements. Reducing felling intensity will not by any means ensure the recovery of these species, but merely hasten the natural decline of their populations [15].

Our results suggest that increasing felling intensity to increase light in the stands and stimulate the growth and survival of future crop trees (as proposed in [8]) is feasible, but should not exceed four trees ha^−1^: beyond this number, biomass recovery might be delayed. Such felling intensity should target the largest possible number of species in order to limit the pressure on any single species. However, there is little chance that the same number of crop trees will be found again in 30 years' time. Our results further highlighted that thinning did increase both gain in biomass and growth and survival of future crop trees in all diameter classes less than 70 cm DHB, while limiting floristic shifts towards more fast-growing pioneers [22]. Although this will have no effect on the timber stock at the end of the first felling cycle, more harvestable trees should be available at the end of the second one. Spreading harvesting of potential timber stock (the ‘primary forest premium’) over at least two cycles, e.g. by increasing diameter cutting limits, while implementing thinning might thus be a promising way towards sustainability.

Such operations, increasing the intensity of disturbance in managed forests, should be limited to productive forests: there is evidence that all forests are not as productive as those in M'Baïki [28,45,46] and should thus be managed differently. This would entail improving the characterization of the forests of the region in terms of floristic/functional composition, long-term history of disturbance and soil potentiality. It might also involve new incentives for logging companies to counter their financial short-term perspective.
